# Increasing the safety and efficacy of chimeric antigen receptor T cell therapy

**DOI:** 10.1007/s13238-017-0411-9

**Published:** 2017-04-22

**Authors:** Hua Li, Yangbing Zhao

**Affiliations:** 1 Center for Cellular Immunotherapies, Perelman School of Medicine, University of Pennsylvania, Philadelphia, PA 19104-5156 USA; 2 Cancer Center, Chengdu Military General Hospital, Chengdu, 610083 China

**Keywords:** chimeric antigen receptors, cancer adoptive immunotherapy, T lymphocytes, gene therapy, gene editing

## Abstract

Chimeric antigen receptor (CAR) T cell therapy is a promising cancer treatment that has recently been undergoing rapid development. However, there are still some major challenges, including precise tumor targeting to avoid off-target or “on-target/off-tumor” toxicity, adequate T cell infiltration and migration to solid tumors and T cell proliferation and persistence across the physical and biochemical barriers of solid tumors. In this review, we focus on the primary challenges and strategies to design safe and effective CAR T cells, including using novel cutting-edge technologies for CAR and vector designs to increase both the safety and efficacy, further T cell modification to overcome the tumor-associated immune suppression, and using gene editing technologies to generate universal CAR T cells. All these efforts promote the development and evolution of CAR T cell therapy and move toward our ultimate goal—curing cancer with high safety, high efficacy, and low cost.

## INTRODUCTION

Immunotherapy has become a promising novel cancer treatment and is entering a new era of rapid growth. Different from traditional therapies for cancer patients, such as surgery, chemotherapy and radiotherapy, immunotherapy is based on the knowledge of the basic mechanisms of the immune system and anti-tumor immune response and aims to harness the immune system to eliminate tumors specifically and effectively. Unlike monoclonal antibody-targeted therapies, such as trastuzumab, bevacizumab, rituximab and cetuximab, which are already used clinically, T cell-based therapy uses genetically modified living autologous or allogeneic immune cells as a drug to rally the patient’s own immune system to destroy cancer cells and has revolutionized the concept of drugs. In contrast to the specific binding of monoclonal antibody drugs to the target, T cells have the capacity to proliferate and lyse cancer cells directly. Furthermore, T cells have the effector and/or memory functions of the normal immune system. Such memory of anti-cancer ability can be maintained for a much longer time in the patient’s body than traditional drugs.

Recent breakthroughs in T cell-based immunotherapy have already yielded exciting results from clinical trials using the adoptive transfer of autologous tumor-infiltrating lymphocytes (TILs) as well as T cells introduced with genetic material encoding a T cell receptor (TCR) or chimeric antigen receptor (CAR). The design of a CAR links antibody-mediated recognition of tumor-associated antigens with the cytotoxic activities of immune effector cells directly. Both CAR- and TCR-redirected T cells can recognize antigen and be activated to be effector cells, but each strategy is distinct and has unique advantages and disadvantages.

TCR-redirected T cells recognize the antigen in a human leukocyte antigen (HLA)-dependent manner, while CAR T cells can be activated directly via CARs in an HLA-independent manner. When CD8^+^ CAR T cells are activated via CARs, they deliver granzyme B and perforin-mediated cytotoxicity as normal cytotoxic T lymphocytes (CTLs). CD4^+^ CAR T cells can also be activated via CARs in an HLA-independent manner to become helper T (Th) cells, secreting cytokines to regulate the immune response. Considering that activated CAR^+^CD4^+^ T cells can provide support to CAR^+^CD8^+^ T cells, CD4^+^ T and CD8^+^ T cells are usually mixed, genetically modified with CAR and then infused into patients in clinical trials. This design boosts cellular immunity and overcomes the HLA restriction and some tumor escape mechanisms, showing exciting potential for broad clinical applications. However, CAR T cell therapy is highly complex. There are numerous unanswered questions, such as how to safely target tumors specifically, how the T cell and tumor interact, how to make T cells fight with the tumor microenvironment efficiently, how to optimize the T cell gene transfer, and how to standardize the production procedure and control the cost of cell manufacture for clinical use. In this review, we will describe the main barriers that remain in eliciting an effective immune response against tumor cells to cure cancer with fewer side effects and the strategies of CAR T cell immunotherapy that will help to overcome these challenges and reach the ultimate goal of curing cancer patients.

## DESIGN OF CAR CONSTRUCTS

**Figure 1:**
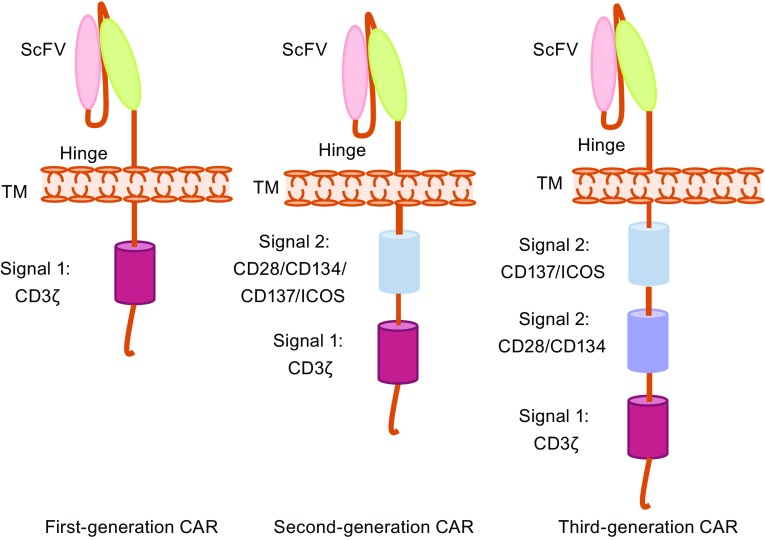
**CAR design**. CARs are engineered membrane proteins that consist of three main components: an extracellular antigen-recognition domain, a hinge and transmembrane domain, and an intracellular T cell activation signaling domain. First-generation CARs have only a CD3ζ signaling domain in the intracellular part providing signal 1 for T cell activation. Second-generation CARs have an extra signal 2 domain in the intracellular domain. Third-generation CARs have three domains in the intracellular part, two signal 2 domains and one signal 1 domain. ScFv, single chain variable fragment; ICOS, inducible T cell costimulator; TM, transmembrane

CARs are engineered membrane proteins that consist of three main components: an extracellular antigen-recognition region, a hinge and transmembrane region, and an intracellular T cell activation region. The antigen-recognition part consists of a single chain variable fragment (scFv) derived from hypervariable regions of an antibody’s immunoglobulin heavy and light chains or an antigen-binding portion linked to a flexible hinge region derived from a CD8 molecule or other molecules, such as the CD28 or Fc region of an antibody. A transmembrane is derived from CD8 or CD28. The intracellular signaling domain is designed for the activation of T cells and is usually derived from the cytoplasmic portion of the CD3ζ chain (Fig. 1).

The first-generation CARs have only the CD3ζ signaling domain, which provides only signal 1 for T cell activation (Eshhar et al., 1993). However, efficient T cell activation needs not only signal 1 but also signal 2 provided from costimulatory molecules (also termed co-stimulatory signals). Early clinical trials using first-generation CARs to treat cancers showed very limited responses and the CAR-modified cells persisted at low levels for only weeks-to-months (Kershaw et al., 2006; Lamers et al., 2006b; Till et al., 2008), suggesting first-generation CAR T cells lack sufficient activation signals to support the long-term T cell expansion that is required for efficient anti-tumor effects.

Second-generation CARs were generated by adding a signaling domain derived from a co-stimulation molecule, such as CD28, 4-1BB (CD137), ICOS or OX40 (CD134), to the intracellular domain of a CAR to provide additional T cell stimulation and activation signals (Maher et al., 2002) (Fig. 1). The second-generation CAR T cells showed an improved capability to expand and persist *in vivo*, an activity that has been demonstrated in clinical trials (Porter et al., 2011). Based on second-generation CARs, third-generation CARs contain multiple costimulatory domains in the intracellular signaling domains (Carpenito et al., 2009; Zhao et al., 2009). However, the superior efficacy has not been validated clinically (Till et al., 2012). Currently, most of the ongoing clinical trials are using second-generation CARs to explore the safety and efficacy of this approach.

## SELECTING STRATEGIES FOR CANDIDATE TARGETS

It remains a great challenge to boost a potent cellular immune response to eradicate not only blood cancers but also solid tumors with tolerable side effects. The most important hurdle is to identify appropriate target antigens, which is a key selection with regard to safety. Thus, ideally, CAR T cells should target strictly tumor cells and spare healthy tissue cells. Except for antigens arising from gene mutations, tumor-associated antigens (TAAs) that are unique to tumor cells are not easy to identify. Because tumors are derived from normal cells, most of the TAAs identified are self-antigens, and such TAAs are usually differentially overexpressed in tumor cells and are also expressed at a low level in some normal cells. What makes the situation more challenging is that immune responses triggered by CAR T cells are much more sensitive than those triggered by antibodies. Moreover, when encountering antigens, the infused CAR T cells can be activated and expanded by over one hundred- to thousand-fold to cause more severe toxicities, such as what has already been reported for the high-affinity HER2/neu (ErbB2) CAR derived from the humanized monoclonal antibody trastuzumab to treat a colon cancer patient (Morgan et al., 2010). The treatment led to the patient’s death due to a large number of administered cells localizing to the lung immediately following infusion, which triggered CAR T cells to release cytokines by the recognition of low levels of HER2 on lung epithelial cells. Therefore, prudent selection strategies for candidate targets are the most important not only for safety but also for the efficacy of CAR T therapy as well.

CD19, a validated target that is widely used in CAR T immunotherapy for CD19-positive hematologic malignancies, is actually not a TAA but is an ideal target. CD19 is a biomarker for normal B cells and neoplastic B cell malignancies that is not expressed in other healthy tissues. CD19 CAR T cells have been extensively applied in clinical trials to treat B cell acute lymphoblastic leukemia (B-ALL), chronic lymphocytic leukemia (CLL), follicular lymphoma (FL), diffuse large B cell lymphoma (DLBCL), B cell non-Hodgkin lymphoma (B-NHL), and mantle-cell lymphoma (MCL). The side effect of successful CD19-CAR T cell therapy is on-target/off-tumor toxicity of B cell aplasia, which is correlated with CD19 CAR T persistence and disease regression. Fortunately, B cell aplasia can be effectively treated by immunoglobulin infusion as a replacement therapy. To minimize the effect on ‘bystander’ healthy B cells, Faitschuk et al. found that the IgM Fc receptor (FcμR) is better than CD19 as a promising candidate target because of its high and consistent overexpression on CD19^+^CD5^+^ CLL cells and considerably lower level of expression on nonmalignant CD19^+^ B cells or other hematopoietic cells. The anti-FcμR CAR T cells showed more specificity and selectivity than anti-CD19 CAR T cells in an animal experiment, suggesting that anti-FcμR CAR T cells may have a superior therapeutic index (Faitschuk et al., 2016). However, both the safety and efficacy of using anti-FcμR CAR T cells to treat CLL require further clinical trial validation.

To treat patients with CD19-negative B cell leukemia or to prevent the relapse of CD19 CAR T treatments, additional targets are explored. CD22, an activation marker of mature B lymphocytes that is expressed on B cell malignancies, with a tissue distribution similar to CD19, was selected as an alternative to CD19. CD22 has been validated as a successful target for B cell leukemia and lymphomas using an immunotoxin approach (FitzGerald et al., 2011). It was reported that targeting a membrane-proximal domain of CD22 is the key element in developing a highly active CD22 CAR (Haso et al., 2013). A clinical trial of CD22 CAR T cell-treated patients, including children, with recurrent and refractory ALL, FL, NHL, or large cell lymphoma (LCL) is underway at the National Cancer Institute (NCT02315612). To effectively control the recurrence of CD19-positive B cell hematologic malignancy, the ongoing clinical trial (NCT02903810) infuses both CD19 and CD22 CAR T cells into the patients with refractory or recurrent CD19+/22+ B-lineage leukemia/lymphoma.

CD20 is a pan-B cell marker expressed on all B cells from the pre-B phase to mature memory B cells. Anti-CD20 monoclonal antibody (Rituximab) therapy has been successfully used to treat indolent B cell non-Hodgkin’s lymphoma (NHL) (Smith, 2003), as well as CLL (Watanabe et al., 2015). In a recent report of a phase IIa clinical trial treating patients with relapsed or refractory B cell non-Hodgkin lymphoma using CD20 CAR T cells, eleven patients were enrolled, and seven patients underwent cytoreductive therapy to debulk the tumors and deplete the lymphocytes before receiving T cell infusions. The overall objective response rate was 81.8% (9 out of 11), with 6 complete remissions and 3 partial remissions; no severe toxicity was observed (Zhang et al., 2016). To prevent the high rate of B cell malignancy relapse, single-chain bispecific CAR T cells against target cells expressing either CD19 or CD20 were designed recently. This CD19-OR-CD20 CAR has OR-gate activity, providing an effective solution to the challenge of antigen escape in CD19 CAR T cell therapy (Zah et al., 2016). Other promising targets that have been developed and are under clinical trial validation to treat hematological malignancies are CD33 and CD123 for acute myeloid leukemia (AML), CD133 for AML and ALL, CD138 and BMCA for multiple myeloma (MM), the immunoglobulin Kappa chain (Igκ) for CLL, and inactive tyrosine-protein kinase transmembrane receptor (ROR1) for CLL and ALL (Carpenter et al., 2013; Finney et al., 1998; Hudecek et al., 2010; Mardiros et al., 2013; Vera et al., 2006; Zhu et al., 2015).

CAR-T cell activation and expansion *in vivo* can lead to the release of toxic levels of cytokines, referred to as cytokine release syndrome (CRS). A subset of patients treated with CD19 CAR T cells develops clinically significant CRS. In many patients, the CRS is mild and patients present with flu-like symptoms, including fever, myalgia, fatigue, and headache. In contrast, other patients develop more fulminant CRS with multisystem organ failure. Recent data demonstrate that IL-10, IL-6, and IFN-γ are the most highly elevated cytokines in patients who develop CRS after CD19 CAR T treatment. It was reported that IL-6 is highly elevated in these patients and temporally correlates with maximum T-cell activation/proliferation (Barrett et al., 2014). Tocilizumab is a recombinant humanized monoclonal antibody against the IL-6R that prevents IL-6 from binding to membrane-bound and soluble IL-6R (Singh et al., 2011). A single dose of the IL-6 receptor antagonist tocilizumab led rapid, dramatic, and complete resolution of life-threatening CRS resulting from CD19 ACR T therapy (Grupp et al., 2013). Other approaches that could be considered include the use of corticosteroids or inhibitors of IL-2R (CD25), IL-1R, or TNF-α (Barrett et al., 2014). However, it is still a challenge to control the toxicity without interfering with efficacy. Current data suggest tocilizumab is effective at reversing CRS without inhibiting the efficacy of CAR T treatment. Further studies are needed to pursue other options.

Until now, most of the reported clinical trials utilizing CAR T cells to treat solid tumors have been far less promising than those used to treat hematological malignancies. The less satisfactory outcomes of the early reported CAR T clinical trials for solid tumors were primarily due to the use of first-generation CARs or on-target/off-tumor toxicities (Lamers et al., 2006a; Linette et al., 2013; Morgan et al., 2013; Parkhurst et al., 2011). In addition, there are other barriers that limit CAR T treatment in solid tumors, among which the most important issues are tumor-suppressive microenvironments, tumor-associated immune suppression, and the sub-optimal quality and quantity of the infused CAR T cells. Neuroblastoma patients with high-risk disease have very poor outcomes despite intensive therapy. Certain antigens that are derived from embryonic neuroectoderm but that are not widely expressed in non-embryonic tissues provide several optional targets for CAR T cell immunotherapy, such as the L1-cell adhesion molecule (L1-CAM/CD171) (Hong et al., 2014; Park et al., 2007)), disialoganglioside (GD2) (Suzuki and Cheung, 2015), O-acetyl-GD2 ganglioside (OAcGD2) (Alvarez-Rueda et al., 2011), and B7H3. GD2 is a well-characterized neuroblastoma antigen that is also expressed on osteosarcomas, and some other sarcomas. A promising clinical trial was reported by Louis et al. in which 19 patients with high-risk neuroblastoma were treated. Eight were in remission at infusion, and 11 had active disease, among whom three patients with active disease achieved complete remission (Louis et al., 2011). However, it is unclear whether the three patients with complete remission solely arose from the GD2 CAR T treatment, due to the fact that those patients also received other treatments after they were treated with the CAR T cells. Other ongoing clinical trials using anti-GD2 CAR T cells for relapsed or refractory neuroblastoma, sarcoma, osteosarcoma, and melanoma are being conducted at different institutions to further validate the safety and efficacy of this treatment.

HER2 is one of the most extensively studied targets for cancer therapy. HER2 is over-expressed in a broad range of malignancies, including brain tumors, sarcomas, breast cancer, lung cancer, and colon cancer. Trastuzumab is an antibody against the extracellular domain of HER2 and is therapeutically active in HER2-overexpressing breast cancers. Severe adverse effects (SAEs) developed in the first clinical trial using CAR T targeting HER2 to treat metastatic colon cancer using a 3rd generation trastuzumab-derived CAR (Zhao et al., 2009). The SAE was caused by targeting HER2 with high-affinity CAR T cells that led to severe toxicity due to target recognition on normal cardiopulmonary tissue (Morgan et al., 2010). Since HER2 is a very attractive target for a broad range of solid tumors, further research and development can potentially define a strategy for a CAR to target HER2 safely and efficiently, such as the use of affinity-tuned scFv, which will be discussed below.

The first clinical trial of treating cancer patients using CAR T was the treatment of ovarian cancer by targeting the a-folate receptor (FRα). The trial showed acceptable safety but no objective response, probably due to the use of a 1st generation CAR and poor penetration into slide tumor and low persistence of transferred T cells (Kershaw et al., 2006). New CARs targeting FRα were developed and showed focused antitumor activity with a reduced potential of toxicity in pre-clinical tumor models (Lanitis et al., 2013). EGFRvIII could be considered a safe tumor target because it is a mutated form of EGFR expressed on glioblastoma multiforme (GBM) tumors (Johnson et al., 2015; Sampson et al., 2014). A pilot study of T cells redirected to EGFRvIII with a CAR in patients with EGFRvIII^+^ glioblastoma was reported at the 2016 ASCO Annual Meeting. The trial showed that EGFRvIII CAR T cells were safe, without evidence of off-target toxicity or cytokine release syndrome, and were immunologically active.

In addition to antigen expressed on the surface of tumor cells, some TAAs on tumor stromal cells come into the selection range of candidate targets. The vascular endothelial growth factor receptor 2 (VEGFR2) is overexpressed in the tumor vasculature. Chinnasamy et al. showed that VEGFR2 CAR T cells have an antitumor effect via effectively targeting and destroying VEGFR-2-expressing cells in the tumor vasculature while sparing non-transformed tissues (Chinnasamy et al., 2010). The results of a clinical trial using VEGFR-2 CAR T cells were disappointing, showing that among 24 recruited patients, none demonstrated a complete response (CR) and only one patient had a partial response (PR). Most of the other patients had progressive disease (PD).

Apart from the membrane protein antigens, Posey et al. proposed that glycopeptides can be used as tumor targets for CAR T cells. CARs targeting the cancer-associated Tn-glycoform of the membrane mucin MUC1 were constructed and demonstrated that they had target-specific cytotoxicity in mice with leukemia and pancreatic cancer (Posey et al., 2016). Because this cancer-associated Tn glycoform of MUC1 is an abnormal self-antigen, with expression restricted to only various cancers and not in normal tissues, it has great potential to be tested in future clinical trials.

Tumor-specific mutated proteins (neoantigens) are another class of tumor antigens, which arise via mutations that alter amino acid coding sequences. Some of the altered amino acid peptides can be expressed, processed, and presented on the cell surface by MHC molecules and subsequently recognized by T cells. Neoantigens represent the ideal targets for cellular immunotherapy because they are not only highly tumor specific but could be very immunogenic because normal tissues do not possess these somatic mutations and neoantigen-specific T cells are not subject to central and peripheral tolerance, and also lack the ability to induce normal tissue destruction (Lu and Robbins, 2016; Schumacher and Schreiber, 2015; Yarchoan et al., 2017). Current strategies of targeting neoantigens for cancer treatment are all based on TCRs that recognize mutant peptide/MHC complexes, because most of the neoantigens identified are expressed inside the tumor cells and processed and presented by MHC molecules (Gubin et al., 2014; Robbins et al., 2013; Tran et al., 2014). Therefore, it is not feasible to target neoantigens by standard CARs that can only recognize cell surface antigens, extracellular TAAs.

To further expand the candidate targets beyond extracellular TAAs, the scFv domain of CAR T cells can be designed to recognize a peptide-MHC complex (Oren et al., 2014; Reiter et al., 1997), enabling such T cell receptor-mimic (TCRm) CAR T cells to recognize a new universe of additional promising intracellular TAAs. One example of an intracellular TAA to be targeted by a CAR is Wilms Tumor 1 (WT1). WT1 is an oncogenic, zinc finger transcription factor that is limited to low levels in the gonads, kidney, spleen, and bone marrow and is overexpressed in hematological malignancies including AML, mesothelioma, gastrointestinal cancer, glioblastoma, and ovarian cancer. Rafiq et al. generated a TCRm CAR against WT1 utilizing the scFv against the WT1/HLA-A*02:01 complex by screening a phage display library. WT1 TCRm CAR T cells demonstrated *in vivo* anti-tumor efficacy and further enhanced proliferation and cytotoxicity when modified to co-deliver the pro-inflammatory IL-12 cytokine (Rafiq et al., 2016). The development of TCRm CARs overcomes the limitation of standard CARs that can only target extracellular TAAs. Because TCRm CARs are designed to mimic TCR in recognizing exactly the same peptide/MHC complex as natural TCR and T cells with natural TCR are precisely selected by the positive and negative selection mechanism in the thymus and peripheral lymphoid organs to avoid autoimmunity, before such TCRm CARs are used clinically, extensive specificity and off-target toxicity investigations are critically important. Moreover, side-by-side comparison of TCRm CARs with relevant TCRs, in both pre-clinical and clinical studies will provide a wonderful chance to further understand the differences between artificial receptors (CARs) and natural TCRs, which is important for the further development of more potent T cell therapies.

## MINIMIZING “ON-TARGET/OFF-TUMOR” TOXICITY

**Figure 2:**
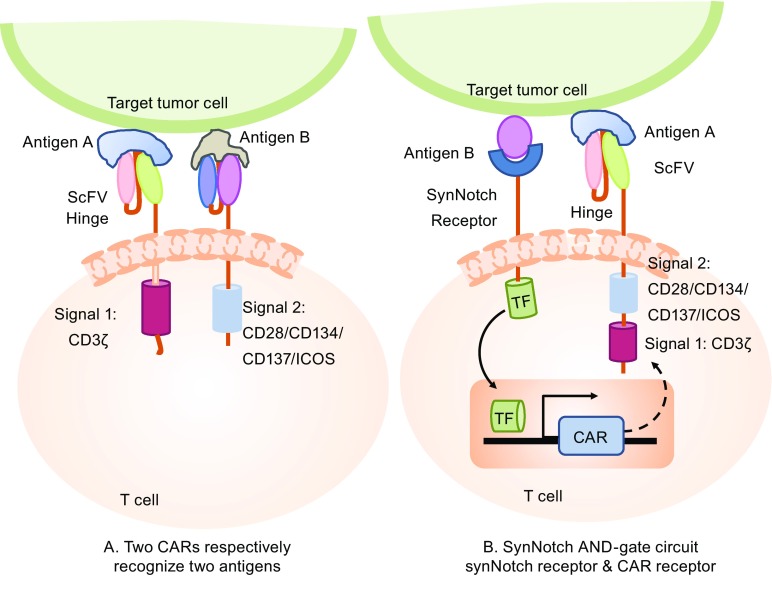
**Antigen A AND antigen B combinatorial antigen recognition to optimize the specificity of CAR T recognition and reduce the “on-target/off-tumor” toxicity**. (A) Two-CAR design strategy: one CAR molecule for activation and another CAR molecule for costimulation in the same T cell. (B) SynNotch AND-gate circuit strategy: the synNotch receptor recognizes antigen B first, then induces the expression of CAR receptor, which recognizes antigen A, and finally activates the T cell. SynNotch, synthetic Notch receptor, which recognizes a target antigen B, then cleaves the receptor to release a transcriptional activator domain to enter the nucleus and induce the expression of CAR; ScFv, single chain variable fragment; ICOS, inducible T cell costimulator; TM, transmembrane

The risk of on-target/off-tumor toxicity is a major obstacle for the development of effective CAR T cell therapies. Strategies to minimize the “on-target/off-tumor” toxicity by improving the specific tumor recognition have to be developed, especially for targeting TAAs that are self-antigens and differentially expressed by tumor cells. One strategy is combinatorial antigen recognition through recognizing two different antigens on the tumor cells via engineering T cells with two CAR molecules: one CAR provides only the activation signal (the same as the first-generation CAR) and the other CAR provides co-stimulation (similar to the second-generation CAR but only has a co-stimulatory moiety, not an activation moiety). Therefore, optimal and effective T cell activation can only be achieved by the recognition of two different antigens simultaneously on the tumor cells, while sparing normal tissue that may only express one of these two antigens (Fig. 2A). Based on this design rationale, T cells expressing two CARs, such as anti-HER2-CD3ζ (signal 1)/anti-MUC1-CD28 (signal 2) (Wilkie et al., 2012) and anti-CD19-CD3ζ (signal 1)/anti-PSMA-CD28-4-1BB (signal 2) (Kloss et al., 2013) were tested in different pre-clinical mouse models. However, there are some concerns regarding using this strategy: the first is the safety concern for the signal 1 CAR recognition antigens expressed normal tissues, because SAE has been reported using 1st-generation CAR (Lamers et al., 2006a); the second concern is increased tumor escape due to the requirement of two antigen recognitions of the tumor cells, resulting in more tumor cells that cannot be effectively targeted due to the heterogeneity of antigen expression patterns of most cancers. Therefore, the feasibility of using this strategy, in terms of both efficacy and safety, requires further validation in both pre-clinical studies and clinical trials.

Recently, an ingenious CAR design, which can achieve “antigen A AND antigen B” combinatorial antigen recognition, has been reported. The design strategy is that the synthetic Notch receptor for one antigen (antigen B) induces the expression of a CAR for a second antigen (antigen A). Thus, the “AND-gate” CAR T cells have a combinatorial antigen recognition circuit working in a sequential manner: the constitutively expressed synthetic Notch (synNotch) receptor first binds with antigen B and then induces CAR molecule expression to recognize antigen A (Fig. 2B). “AND-gate CAR T” cells with the synNotch receptor have the same anti-tumor efficacy as regular CAR T cells, with much higher target cell discrimination. Moreover, the synNotch receptors can also be used to sculpt custom-response programs in primary T cells: they can drive a la carte cytokine secretion profiles, biased T cell differentiation, and local delivery of non-native therapeutic payloads in response to antigen. (Roybal et al., 2016a, b).

Most of the validated TAAs are preferentially up-regulated or over expressed in tumors but are expressed in some normal tissues at low levels, such as HER2 and EGFR. CAR with high-affinity scFv targeting HER2 was used in a clinical trial and led to treatment-associated death due to SAE resulting from on-target/off-tumor toxicity. Evidence has shown that the scFv of the CAR molecule with very high affinity is inversely correlated with the T cell activation threshold and does not necessarily improve antitumor efficacy (Chmielewski et al., 2004; Hudecek et al., 2013). We and other groups have demonstrated that decreasing the affinity of HER2 or EGFR CARs could significantly increase the therapeutic index of CAR T cells *in vitro* and in xenogeneic mouse tumor models (Caruso et al., 2015; Liu et al., 2015). A clinical trial using a HER2 CAR composed of a lower affinity scFv from antibody FRP5 has been reported. Nineteen patients with HER2/Neu-positive tumors (16 osteosarcomas, one Ewing sarcoma, one primitive neuroectodermal tumor, and one desmoplastic small round cell tumor) were treated with the HER2 CAR T cells with the T cell dose ranging from 1 × 10^4^/m^2^ to 1 × 10^8^/m^2^. The CAR T cells persist for up to 6 weeks without evidence of toxicities (Ahmed et al., 2015). A clinical trial reported by Feng et al. used a CAR against EGFR with an appropriate affinity to treat 11 advanced relapsed/refractory non-small cell lung cancer patients with a median dose of 0.97 × 10^7^ cells·kg^−1^ CAR^+^ T cells, with two partial response and 5 stable disease outcomes. This study demonstrated that the EGFR CAR T protocol is safe and feasible for treating EGFR-positive advanced relapsed/refractory NSCLC (Feng et al., 2016). These two clinical trials suggest that T cells engineered with properly selected CARs targeting HER2 or EGFR could be safely used in cancer patients with high HER2 or EGFR expression.

**Figure 3:**
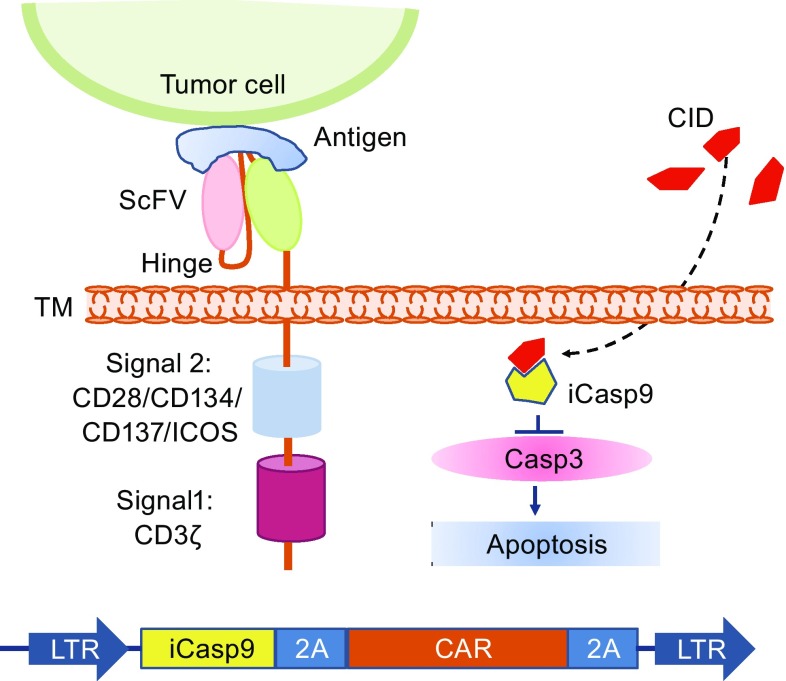
**Armed CAR T cell with “OFF switch,” suicide gene—iCasp9 to kill itself by apoptosis**. The iCasp9 gene and CAR gene are linked by the 2A sequence in the CAR vector, and therefore, iCasp9 is co-expressed with the CAR molecule in CAR T cells. Once the small molecule dimerizer chemical inducer of dimerization (CID) AP20187 is added, it binds to iCasp9, triggering apoptosis. iCasp9, inducible suicide gene caspase-9; Casp3, caspase-3; ScFv, single chain variable fragment; ICOS, inducible T cell costimulator; LTR, long terminal repeats; TM, transmembrane

**Figure 4:**
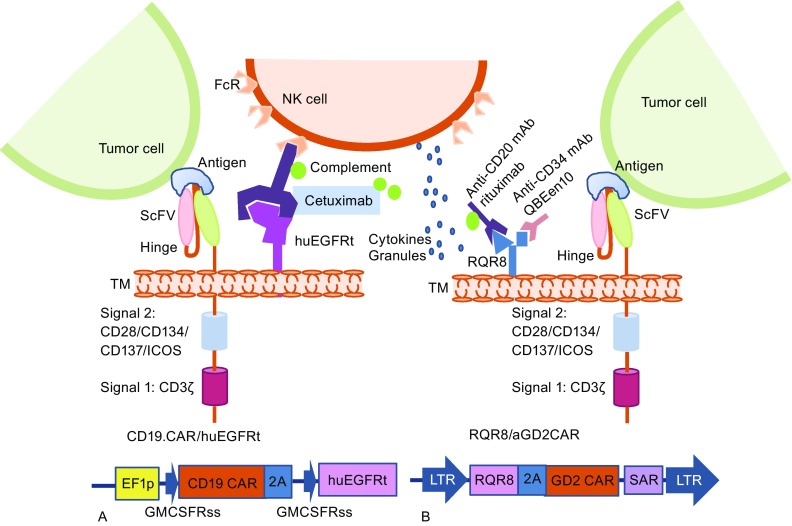
**CAR T cell with “OFF switch”—co-expression with a truncated protein or a small epitope peptide**. In the presence of the mAb of the anti-epitope/truncated protein, the mAb binds the truncated protein or epitope peptide and mediates antibody-dependent cell-mediated cytotoxicity (ADCC) or complement-dependent cytotoxicity (CDC) to trigger cell lysis. The design strategy of an AND logic gate that requires “antigen + mAb”. NK cell, natural killer cells; FcR, Fc receptor; cetuximab, a chimeric monoclonal antibody against epidermal growth factor receptor; huEGFRt, a truncated human EGFR polypeptide; ScFv, single chain variable fragment; ICOS, inducible T cell costimulator; TM, transmembrane; EF1p, elongation factor 1 promoter sequences; GMCSFRss, the GM-CSF receptor-α chain signal sequences; RQR8, a 136-amino-acid marker with epitopes for CD34 and CD20 as a suicide molecule. SAR, scaffold attachment region

To control CAR T associated toxicities, introducing suicide genes into CAR T cells has been under evaluation in clinical trials using herpes simplex virus thymidine kinase (HSV-TK) or inducible caspase-9 (iCasp9) genes (Bonini et al., 1997; Ciceri et al., 2009; Oliveira et al., 2012). iCasp9 has been clinically validated in preventing graft-versus-host disease (GVHD) in hematopoietic stem cell transplantation patients. Hoyos et al. combined the suicide gene with CAR T and generated a retroviral vector bearing CD19 CAR, IL-15, and iCasp9 genes. The iCasp9 suicide gene can induce apoptosis upon specific binding with a chemical inducer of dimerization (CID) AP20187 (Fig. 3). The CD19 CAR/IL-15/iCasp9 T cells have enhanced expansion and antitumor activity *in vivo* and can be eliminated by exposure to CID, further increasing the safety of the proposed approach (Hoyos et al., 2010). Another approach used to ablate CAR T cells is to co-express on the T cell a truncated protein recognized by clinically approved mAbs, and thus, the transduced T cells could be eliminated by antibody-dependent cellular cytotoxicity (ADCC) or complement-dependent cytotoxicity (CDC) after the administration of the relevant mAb. The truncated protein could be a polypeptide of CD molecules or an epitope of mAbs, such as a truncated human EGFR polypeptide (huEGFRt) (Fig. 4) (Wang et al., 2011). Considering the heavy genetic payload of a combined marker and suicide gene together with a gene vector, Philip et al. developed a highly compact epitope, RQR8, which can be recognized not only by anti-CD34 mAb QBEnd10, the antibody used in the Miltenyi CliniMACS CD34 selection system, but also by the anti-CD20 antibody rituximab. Therefore, RQR8 serves as both a selection marker by binding with CD34 and a suicide molecule by binding with rituximab, which induces ADCC and CDC. This RQR8 epitope-based marker/suicide gene system was designed to match the Miltenyi CliniMACS system and took a step forward to the safer and inexpensive clinical use of CAR T cells (Philip et al., 2014).

**Figure 5:**
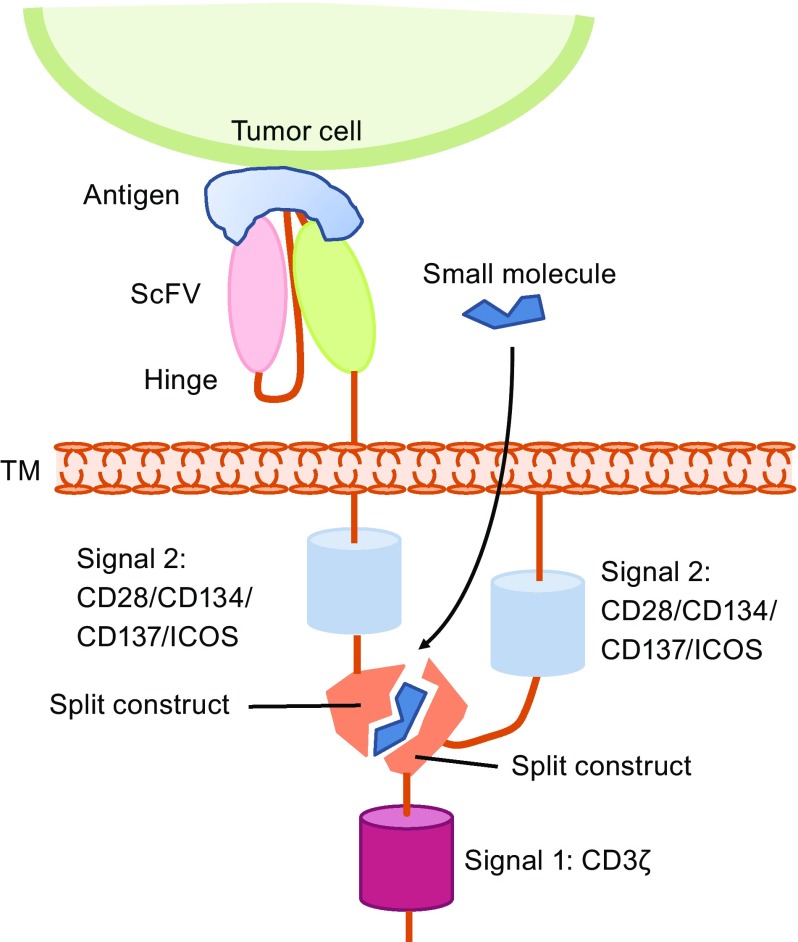
**CAR T cells with “ON switch”—small molecules**. Only when the small molecule is present can two parts of CAR be reassembled with the split construction to activate CAR T cells. The design strategy of an AND logic gate that requires “antigen + small molecule” together for T cell activation. ScFv, single chain variable fragment; ICOS, inducible T cell costimulator; TM, transmembrane

**Figure 6:**
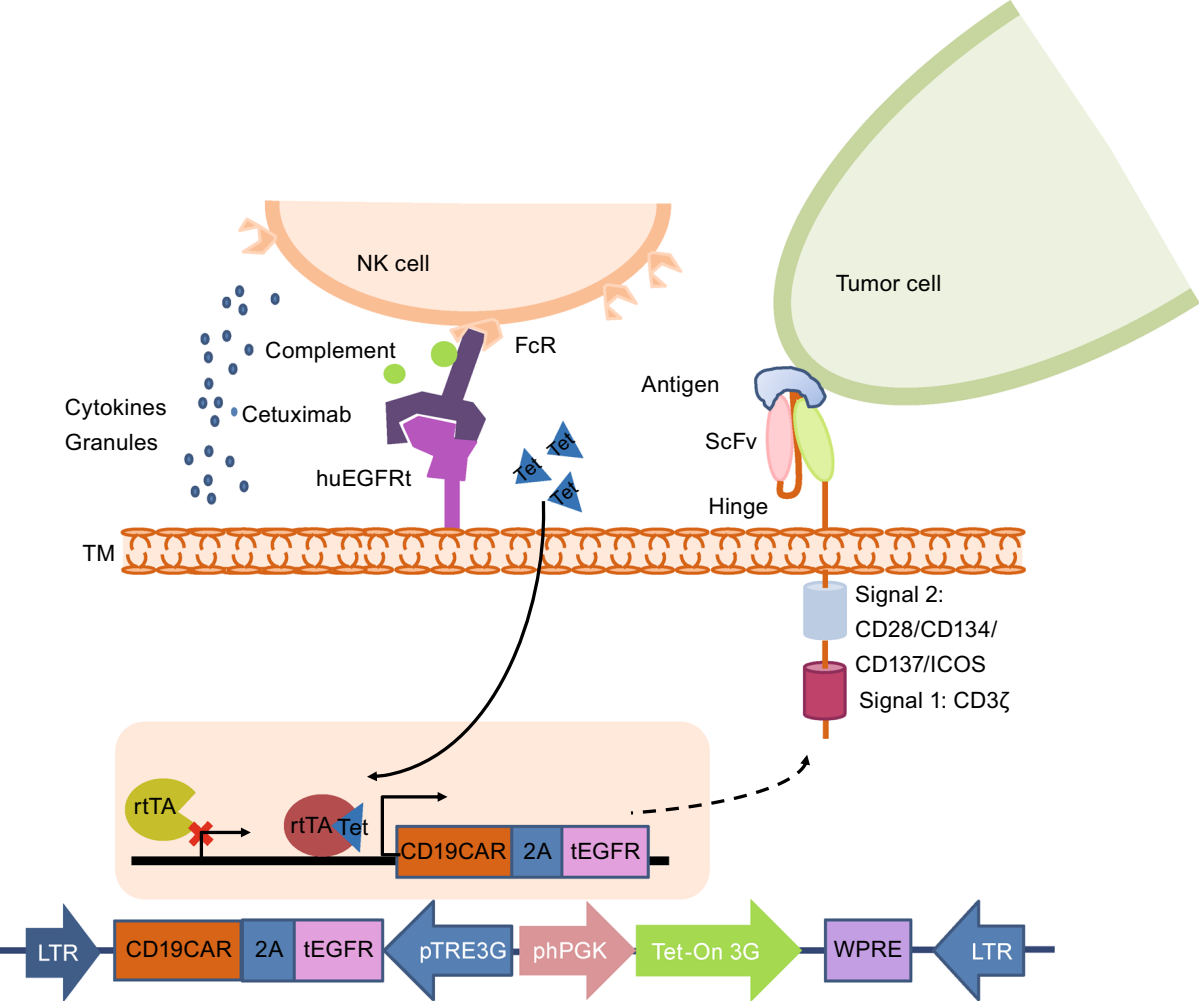
**CAR T cells with “ON/OFF switch”**. The “ON” switch utilizes the Tet-On inducible system. Only when tetracycline (Tet) is present, rtTA (r everse t etracycline-controlled t rans a ctivator) binds with Tet and induces the expression of the CAR molecule and marker protein, such as truncated EGFR (tEGFR). The “OFF” switch utilizes mAb-mediated ADCC or CDC. Only when the anti-marker protein Ab (such as cetuximab) is present does the mAb bind with the marker protein and mediate ADCC or CDC to lyse CAR T cells

Another exciting strategy for developing the next generation of precision-controlled therapeutic CAR T cells is the remote “ON-switch” control of CAR T cells that allows physicians to precisely control the timing, location, and dosage of T cell activity by pharmacologic regulation, thereby mitigating toxicity. Wu et al. developed split receptors in which antigen binding and intracellular signaling components assemble only in the presence of a heterodimerizing small molecule (Fig. 5). This design with an AND logic gate needs two requirements for CAR T cell activation: antigen and small molecule. The ON-switch CAR T cells can be effectively controlled with a small molecule *in vivo* and can selectively kill cognate target cells when exposed to the dimerization-inducing molecule. Furthermore, the unrelated dimerization systems can also be used to control the ON-switch CAR architecture (Wu et al., 2015). Controlling the expression and functions of CARs with an inducible system to avoid the toxicities of CAR T cells and improve the safety profile of CAR T immunotherapy was also attempted. Sakemura et al. utilized an all-in-one, third-generation (3G) tetracycline (Tet)-inducible vector and inserted a CD19 CAR into the pRetroX-TetOne 3G vector, allowing the CD19 CAR expression to be controlled by doxycycline (Dox) (Sakemura et al., 2016). In addition to the antitumor function similar to that of conventional CD19 CAR T cells, TET-ON CD19 CAR T cells have two separate statuses, “ON” and “OFF,” controlled by Dox administration, which allows for reversible regulation of CAR expression via the administration of a drug to the patients or stopping on demand in the clinic (Fig. 6).

While CAR T cell immunotherapy has begun to demonstrate efficacy, cell-engineering techniques that result in permanent genomic modification, such as lentiviral or retroviral vectors, carry some safety concerns, such as the potential insertional mutagenesis and malignant transformation of the transduced cells by integrating provirus into the genome. Primary T cells can be easily transferred with *in vitro*-transcribed RNA for high transgene expression transiently without integration-associated safety concerns (Zhao et al., 2006). Treating cancer patients with T cells that are transiently transferred with CARs provides an alternative for a safer and potentially effective therapy. It has been demonstrated that multiple injections of RNA-transferred CAR T cells showed effective antitumor responses in preclinical mouse sloid tumor models (Singh et al., 2014; Zhao et al., 2010). A clinical trial reported using RNA-transferred CAR T cells to treat pancreatic cancer or mesothelioma patients showed feasibility and safety, without overt evidence of off-tumor/on-target toxicity against normal tissues (Beatty et al., 2014). A patient with anaphylaxis and cardiac arrest was reported in the trial, most likely through IgE antibodies specific to the CAR, which used mouse-origin scFv, indicating that the potential immunogenicity of CARs derived from murine antibodies can be a safety issue, especially when CAR T cells are administered using an intermittent dosing schedule (Maus et al., 2013). Side-by-side comparison of single-dose, lentivirus-transduced T cells with multiple-dose infusions of RNA-transferred CAR T cells in pre-clinical mouse tumor models showed less efficient tumor control ability of RNA CAR T cells, partially due to the weaker tumor-penetrating ability of RNA CAR T cells (Singh et al., 2014).

Due to the transient transgene expression, RNA CAR T cells are unable to serve as serial killers that kill and penetrate tumors as efficiently as lentiviral or retroviral vector-transduced CAR T cells. However, T cells were demonstrated to be good antigen-presenting cells, especially when the T cells were activated. A clinical trial reported using mRNA CAR T cells that target mesothelin to treat cancer patients, and broad anti-tumor immune responses against different tumor antigens, other than CAR T-targeted antigen, were detected from two of the four reported patients, indicating multiple injections of RNA CAR T cells could result in epitope spreading, and the infused RNA CAR T cells not only served as tumor killers to directly lyse tumor cells but also served as an adjuvant/vaccine to boost immune responses against cancers (Beatty et al., 2014). Thus, RNA CAR transfection of T cells could be potentially developed into a safe and potent cancer therapy.

## BREAKING THE PHYSICAL AND BIOCHEMICAL BARRIERS OF SOLID TUMORS

Apart from the on-target/off-tumor and off-target toxicities that limit the development of efficient CAR T therapies, there are numerous factors that influence the final outcome of CAR T treatment. It is fundamentally important to generate CAR T cells that are not only safe but also have a high capacity for efficient trafficking/migrating to tumor sites, expanding and persisting after tumor stimulation, as well as with potent tumor-killing ability. For CAR T cells to treat solid tumors, the physical and biochemical barriers established by the tumors to counteract anti-tumor immunity need to be considered as the highest priority because these immunosuppressive factors compose the main obstacle for successful CAR T therapy. Furthermore, for CAR T cells that are infused into the bloodstream of cancer patients, homing to the tumors across physical and biochemical barriers is a crucial requirement for the effectiveness of CAR T cell immunotherapy. The importance has been shown in a phase I study treating ovarian cancer patients, in which CAR T cells were found to be accumulated in a pelvic mass in only one of seven treated patients, and no clinical responses were observed. Whole-body imaging showed that CAR T cells were localized in the lungs, liver,and spleen but not in ovary in all seven patients (Kershaw et al., 2006). The poor trafficking of CAR T cells to the tumors could be potentially overcome by improving the T manufacture quality, further T cell modifications, CAR design/target selection, and preconditioning regimen. As solid tumors usually have a surrounding stroma and an abnormal vasculature that impede the efficient penetration and infiltration of CAR T cells, changing the delivery route from systematic intravenous infusion to local injection to bypass the T cell trafficking from bloodstream into the tumors could potentially achieve better treatment efficacy for some cancer patients. Recently, an inspiring case report showed regression of all intracranial and spinal tumors for a patient with recurrent multifocal glioblastoma who received CAR T cells targeting glioma-associated antigen interleukin-13 receptor alpha 2 (IL13Rα2). Increased levels of cytokines and immune cells in the cerebrospinal fluid showed that this local infusion could effectively elicit an antitumor response (Brown et al., 2016). Efforts have been made to improve CAR T cell homing to tumor targets by co-expressing the chemokine receptor on CAR T cells, such as the co-expression of the chemokine receptor CCR2b, which directs the migration of the T cells toward CCL2 produced by the tumors. Improved CAR T cell homing (>10-fold) to CCL2-secreting neuroblastoma and enhanced antitumor activity *in vivo* were found by the co-expression of CCR2b on GD2 CAR T cells (Craddock et al., 2010).

The tumor-associated hypoxic microenvironment, inhibitory checkpoint molecules, byproducts from the altered metabolism of solid tumors and tumor-induced suppressive cells are the major biochemical barriers that prevent effective CAR T therapy. Among these immunosuppressive factors, inhibitory checkpoint molecules, such as PD-1, cytotoxic T lymphocyte-associated protein 4 (CTLA-4), and T cell immunoglobulin and mucin domain-3 (TIM-3) are well-studied targets for antibody-based immunotherapy. Blocking the binding of immunosuppressive molecules with ligands for specific antibodies significantly enhanced anti-tumor immune responses both in preclinical models (Curran et al., 2010; Sakuishi et al., 2010) and patients (Robert et al., 2015; Taube et al., 2014; Topalian et al., 2014; Wolchok et al., 2013). The clinical successes of antibody-mediated checkpoint blockade in antibody-based immunotherapy have encouraged researchers to combine CAR T therapy with checkpoint antibody blockade. Using an orthotropic mouse model of pleural mesothelioma, Cherkassky et al. demonstrated that PD-L1/PD-L2 within the tumor microenvironment mediated CAR T cell exhaustion. Through combining PD-1 antibody checkpoint blockade with cell-intrinsic PD-1 shRNA blockade or a PD-1 dominant-negative receptor, the antitumor function of CAR T cells could be restored. These findings showed that interfering with PD-1/PD-L1 may be an effective strategy to improve CAR T cell therapy in solid tumors (Cherkassky et al., 2016). A genetic approach in which T cells were transduced with both a CAR and a chimeric switch receptor containing the extracellular domain of PD-1 fused to the transmembrane and cytoplasmic domain of the costimulatory molecule CD28 (PD1-CD28 switch receptor) was proposed (Ankri et al., 2013; Liu et al., 2016a; Prosser et al., 2012). Compared with combining two therapies of CAR T cells and checkpoint inhibitor antibody, using T cells expressing both a CAR and PD1-CD28 switch receptor as a single therapy has the potential benefits of not only both synergizing CAR T with reduced PD1 inhibitory signals and providing T cells with an additional positive costimulatory signal simultaneously but also reducing both the checkpoint inhibitor antibody therapy-associated side effects and the cost of the treatments.

The advantage of co-expressing CAR and PD1-CD28 switch receptor is that the CAR T cells’ anti-tumor potency can be further improved by offsetting the inhibitory signals of PD1/PD-L1/2 and at the same time, providing T cells with additional CD28 costimulatory signal, which was supported by a recent study that tested T cells transferred with CARs and switch receptors in different clinically relevant mouse tumor models for mesothelioma and prostate cancer by targeting mesothelin or prostate stem cell antigen (PSCA). This study showed that the treatment of mice bearing large established solid tumors with CAR T cells co-expressing PD1-CD28 switch receptor led to a significantly improved regression of tumors due to enhanced CAR T infiltration, increased CAR T cell proliferation and persistence, decreased susceptibility to tumor-induced hypofunction and attenuation of inhibitor receptor expression, compared with treatments with CAR T cells alone or CAR T cells with PD1 checkpoint inhibitor (Liu et al., 2016a). These findings suggest that the application of the PD1-CD28 switch receptor to boost CAR T cell activity is efficacious against solid tumors via various mechanisms, prompting the clinical investigation of this potentially promising treatment modality.

Inhibition from byproducts of tumor cell metabolism, such as cyclooxygenase-2 (COX-2), prostaglandin E2 (PGE2), and adenosine activate protein kinase A (PKA), has also attracted much attention. Newick et al. proposed a new strategy of arming CAR T cells with an interrupter to target the byproduct inhibitors in their recent study (Newick et al., 2016). CAR T cells were generated to express a “regulatory subunit I anchoring disruptor” (RIAD) small peptide. Such CAR-RIAD T cells showed enhanced antitumor activity *in vitro* and showed more efficient migration to tumor sites *in vivo* by blocking the association of PKA with ezrin to blunt the inhibition of PKA on TCR activation. (Newick et al., 2016). IL-12 is an important T cell-stimulating cytokine that mediates the enhancement of the cytotoxic activity of NK cells and T cells and reverses the immunosuppressive tumor microenvironment (Tugues et al., 2015). Zhang et al. designed T cells with inducible IL-12 expression under the control of the nuclear-factor of the activated T cell (NFAT)-derived minimal promoter to initiate IL-12 transcription upon T cell activation. They constructed T cells with MART-1 TCR and inducible IL12 vectors and demonstrated that utilizing the immunostimulatory properties of IL-12 can enhance the antitumor activity of TCR-engineered T cells (Zhang et al., 2011). Subsequently, Chmielewski et al. constructed CAR T cells with inducible IL-12 expression and further demonstrated that such CAR TRUCK T cells (T cells redirected for universal cytokine-mediated killing) showed improved antitumor attack and could recruit and activate innate immune cells to mediate an antigen-independent antitumor reaction in the tumor lesion. Based on this rationale, CAR T cells can be engineered together with other immune modifiers, such as IL-2 and IL-18, or costimulatory ligands to shape the tumor environment (Chmielewski and Abken, 2015; Chmielewski et al., 2014). However, a clinical trial in which metastatic melanoma was treated with inducible IL-12-engineered TILs showed that increasing cell doses were associated with high serum levels of IL-12 and gamma-interferon, as well as clinical toxicities (Zhang et al., 2015). Another clinical trial in which metastatic melanoma and metastatic renal cancer were treated with NY-ESO-1 TCR/inducible IL-12-engineered T cells has been terminated (NCT01457131). Further studies are required to improve the strategies to safely use IL-12.

## USE OF GENE EDITING TECHNOLOGIES TO GENERATE UNIVERSAL CAR T CELLS

Most of the current adoptive immunotherapy clinical trials utilize autologous T cells, which can be hampered by the poor quality and quantity of T cells, as well as by the time and expense of manufacturing autologous T cell products. Thus, using genetically engineered allogeneic “universal” T cells could circumvent the limitations of using autologous T cells and could potentially be developed into a next-generation highly efficient CAR T therapy. Such off-the-shelf universal CAR T cells can be generated from healthy donors to treat multiple patients. The major barriers that prevent the successful use of T cells in the allogeneic settings are graft versus host disease (GVHD) and the rejection of the infused allogeneic T cells by the recipients. Because TCRs on allogeneic T cells may recognize the alloantigens of the recipient, leading to GVHD, and the expression of HLA on the surface of allogeneic T cells may lead to the rapid rejection of the allogeneic T cells by the host immune system, simple and efficient gene editing methods are needed for multiplex genomic editing of T cells to ablate the expression of TCRs, MHC, and other molecules.

The zinc-finger nucleases (ZFNs) are engineered endonucleases composed of a tandem array of zinc finger DNA-binding domains, which can be designed to selectively bind a DNA sequence of choice, coupled to the catalytic domain from the type IIS restriction enzyme FokI. ZFNs were first used to knockout endogenous TCRs to improve the safety and function of TCR-transduced T cells (Provasi et al., 2012). Subsequently, Torikai et al. eliminated the endogenous TCR or HLA-A (Torikai et al., 2013) of T cells from healthy volunteer donors using ZFNs to generate TCR^neg^ CD19 CAR T cells or HLA^neg^ CD19 CAR T cells. It was later reported that the immune inhibitory checkpoint molecule PD-1 on tumor-infiltrating lymphocytes (TILs) from patients with metastatic melanoma could be ablated using ZFNs (Beane et al., 2015). Similar to ZFNs, transcription activator-like effector nucleases (TALENs) can be engineered to cut specific sequences of DNA and can be introduced into cells for use in gene editing. TALENs were used to eliminate the expression of the endogenous TCR α and β chains to improve the expression and functionality of transgenic virus-specific TCRs (Berdien et al., 2014). Valton et al. recently reported a procedure of genetic engineering and characterization of CAR T cells with TCRs disrupted and resistant to three different purine nucleotide analogs (PNA) as preconditioning lympho-depleting regimens. The engineering process includes lentiviral transduction for CAR expression followed by simultaneous TALEN-mediated gene processing of the TCR alpha constant region (TRAC) and deoxycytidine kinase (dCK) responsible for TCR/CD3 surface expression and PNA toxicity, respectively (Valton et al., 2015). This study led to a compassionate therapy of two infant B-ALL patients using universal TALEN gene-edited CAR T cells. These two patients received universal CD19 CAR T cells and were found to be in remission by 4 weeks and 1 month respectively. Then, the patients received an allogeneic stem cell transplant. There was no evidence of GVHD and the patients were in molecular remission 12 months after therapy (Qasim et al., 2017).

The clustered regularly interspaced short palindromic repeats (CRISPR) system is a newly developed simple but very versatile and precise gene-editing technique with the unique ability to easily achieve highly efficient multiplex gene editing (Cong et al., 2013). Ren et al. demonstrated the highly efficient multiplex CRISPR/CAS9 gene editing of TCR, beta-2-microglobulin and PD- to generate allogeneic universal T cells resistant to PD-1 inhibition (Ren et al., 2016). Subsequently, Liu et al. also published their study in which double-knockout (B2M and TRAC, DKO) and triple-knockout (B2M, TRAC and PD-1, TKO) CAR T cells were generated by CRISPR/Cas9 (Liu et al., 2016b). Both groups demonstrated that DKO CAR T cells maintained cytotoxic function *in vitro* and antitumor function without GVHD in xenograft mouse tumor models. Ren et al. (2016) also detected that TKO CAR T cells showed significantly improved anti-tumor activity compared with DKO in a leukemia xenograft mouse model. However, the disruption of HLA class I by T cells could bring a risk of being attacked by NK cells from the patients, because NK cells from recipient patients may lyse CAR T cells without HLA loci by “missing-self recognition”.

Solutions to confer NK resistance include the depletion of NK cells with anti-NK cell antibody or engineering other NK inhibitors, HLA-E or HLA-G into CAR T cells in further studies (Torikai et al., 2013). Disruption of HLA class I alone may delay the rejection but may not be sufficient to avoid being rejected eventually by the recipients. Unlike allogeneic organ transplantation or bone marrow transplantation, which require the transplants to survive for life, universal CAR T cells could be infused multiple times as a drug, provided that the functional allogeneic CAR T cells are safe to use and could survive in patients for a sufficiently long period. Furthermore, additional development is required to ensure the potency of this allogeneic CAR T therapy, at least as potent as its autologous counterpart. Pursuing long-term survival of the universal CAR T cells remains important and a priority. Clinical trials and further studies of the mechanism and key molecules that are involved in the rejection of allogeneic universal T cells would help to develop strategies to achieve the goals. The development of proper pre-conditioning regimens and immune-tolerance induction strategies, such as those that have been developed for organs or bone marrow transplantation, would greatly facilitate the successful use of the allogeneic universal CAR T cells.

Recently, a streamlined strategy was reported for generating allogeneic CAR T cells by using a gene editing approach to target the insertion of a CAR expression cassette while simultaneously knocking out the native TCR in activated T cells (Eyquem et al., 2017; MacLeod et al., 2017). It was showed that directing a CD19-specific CAR to the T-cell receptor α constant (TRAC) locus not only results in uniform CAR expression in CD3^−^ T cells, but also enhances T-cell potency, with edited cells vastly outperforming conventionally generated CAR T cells in a mouse model of acute lymphoblastic leukaemia. The work also demonstrated that targeting the CAR to the TRAC locus averts tonic CAR signaling and establishes effective internalization and re-expression of the CAR following single or repeated exposure to antigen, delaying effector T-cell differentiation and exhaustion (Eyquem et al., 2017).

As reported recently that allogeneic “off-the-shelf” CAR T cells were successfully used to treat two infants with relapsed refractory acute lymphocytic leukemia and bridge them to allogeneic stem cell transplantation (Qasim et al., 2017), allogeneic universal CAR T cells could also be used as conditioning regimens for allogeneic hematopoietic cell transplantation (allo-HCT), thus extending the application of the universal CAR T cells, as a counterpart of non-gene edited allogeneic CAR T cell therapy (similar to donor lymphocytes infusion) followed by allo-HCT, which has been tested in the clinic for relapse refractory CLL, ALL and lymphoma, leading to comparable clinical outcomes (Maude et al., 2014; Porter et al., 2015; Schubert et al., 2016).

## CONCLUSIONS

**Figure 7:**
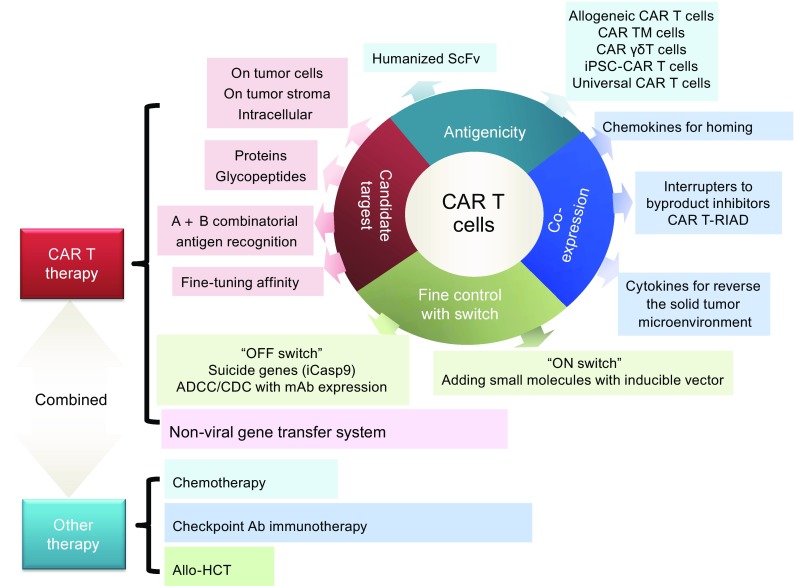
**Strategies for CAR T therapy to overcome challenges in treating cancers**. CAR T therapy is not only a complex technology that requires the optimization of the CAR T cell design but also a clinical application that can be combined with other therapies to achieve high efficacy and safety

CAR T cell therapy is a rapidly evolving field that has shown impressive results in research and clinical trials. However, there are still some major challenges, including precise tumor targeting to avoid off-target or “on-target/off-tumor” toxicity, adequate T cell infiltration and migration to the solid tumor, fine control of CAR T cell activation, proliferation and persistence, side effect management, prevention of genome toxicities of insertional mutagenesis and malignant transformation derived from viral vector transduction and/or gene editing, standardization of manufacture and cost control. Novel cutting-edge technologies for CAR vector design and T cell modification to overcome the hurdles discussed above are very inspiring (Fig. 7). Particularly, we witnessed significant improvements in developing a new generation of CAR T cell therapies. It is with great hope that our growing wealth of knowledge regarding the interaction between the immune system and tumors, together with speedy technological advances, will promote the development of CAR T cell therapy and move toward our ultimate goal—curing cancer with high safety, high efficacy, and low cost.
